# Effect of Natural Polysaccharide Matrix-Based Selenium Nanocomposites on *Phytophthora cactorum* and Rhizospheric Microorganisms

**DOI:** 10.3390/nano11092274

**Published:** 2021-09-01

**Authors:** Alla I. Perfileva, Olga M. Tsivileva, Olga A. Nozhkina, Marina S. Karepova, Irina A. Graskova, Tatjana V. Ganenko, Boris G. Sukhov, Konstantin V. Krutovsky

**Affiliations:** 1 Laboratory of Plant-Microbe Interactions, Siberian Institute of Plant Physiology and Biochemistry, Siberian Branch of the Russian Academy of Sciences, 664033 Irkutsk, Russia; alla.light@mail.ru (A.I.P.); smallolga@mail.ru (O.A.N.); marina.tretjakova@yandex.ru (M.S.K.); graskova@sifibr.irk.ru (I.A.G.); 2 Laboratory of Microbiology, Institute of Biochemistry and Physiology of Plants and Microorganisms, Russian Academy of Sciences, 410049 Saratov, Russia; tsivileva_o@ibppm.ru; 3 Laboratory of Functional Nanomaterials, A.E. Favorsky Irkutsk Institute of Chemistry, Siberian Branch of the Russian Academy of Sciences, 664033 Irkutsk, Russia; ganenko@irioch.irk.ru; 4 Laboratory of Nanoparticles, V.V. Voevodsky Institute of Chemical Kinetics and Combustion, Siberian Branch of the Russian Academy of Sciences, 630090 Novosibirsk, Russia; boris_sukhov@mail.ru; 5 Department of Forest Genetics and Forest Tree Breeding, Faculty of Forest Sciences and Forest Ecology, Georg-August University of Göttingen, Büsgenweg 2, D-37077 Göttingen, Germany; 6 Center for Integrated Breeding Research (CiBreed), Georg-August University of Göttingen, Albrecht-Thaer-Weg 3, D-37075 Göttingen, Germany; 7 Laboratory of Population Genetics, N. I. Vavilov Institute of General Genetics, Russian Academy of Sciences, Gubkin Str. 3, 119333 Moscow, Russia; 8 Laboratory of Forest Genomics, Genome Research and Education Center, Institute of Fundamental Biology and Biotechnology, Siberian Federal University, 660036 Krasnoyarsk, Russia; 9 Forestry Faculty, G. F. Morozov Voronezh State University of Forestry and Technologies, Timiryazev St. 8, 394087 Voronezh, Russia

**Keywords:** arabinogalactan, *Phytophthora cactorum*, *Acinetobacter guillouiae*, *Rhodococcus erythropolis*, *Pseudomonas oryzihabitans*, selenium, nanocomposites, polysaccharides, fungicidal effect, antibacterial activity, potato productivity

## Abstract

We studied the effects of new chemically synthesized selenium (Se) nanocomposites (NCs) based on natural polysaccharide matrices arabinogalactan (AG), starch (ST), and kappa-carrageenan (CAR) on the viability of phytopathogen *Phytophthora cactorum*, rhizospheric bacteria, and potato productivity in the field experiment. Using transmission electron microscopy (TEM), it was shown that the nanocomposites contained nanoparticles varying from 20 to 180 nm in size depending on the type of NC. All three investigated NCs had a fungicidal effect even at the lowest tested concentrations of 50 µg/mL for Se/AG NC (3 µg/mL Se), 35 µg/mL for Se/ST NC (0.5 µg/mL Se), and 39 µg/mL for Se/CAR NC (1.4 µg/mL Se), including concentration of 0.000625% Se (6.25 µg/mL) in the final suspension, which was used to study Se NC effects on bacterial growth of the three common rhizospheric bacteria *Acinetobacter guillouiae*, *Rhodococcus erythropolis* and *Pseudomonas oryzihabitans* isolated from the rhizosphere of plants growing in the Irkutsk Region, Russia. The AG-based Se NC (Se/AG NC) and CAR-based Se NC (Se/CAR NC) exhibited the greatest inhibition of fungal growth up to 60% (at 300 µg/mL) and 49% (at 234 µg/mL), respectively. The safe use of Se NCs against phytopathogens requires them to be environmentally friendly without negative effects on rhizospheric microorganisms. The same concentration of 0.000625% Se (6.25 µg/mL) in the final suspension of all three Se NCs (which corresponds to 105.57 µg/mL for Se/AG NC, 428.08 µg/mL for Se/ST NC and 170.30 µg/mL for Se/CAR NC) was used to study their effect on bacterial growth (bactericidal, bacteriostatic, and biofilm formation effects) of the three rhizospheric bacteria. Based on our earlier studies this concentration had an antibacterial effect against the phytopathogenic bacterium *Clavibacter sepedonicus* that causes diseases of potato ring rot, but did not negatively affect the viability of potato plants at this concentration. In this study, using this concentration no bacteriostatic and bactericidal activity of all three Se NCs were found against *Rhodococcus erythropolis* based on the optical density of a bacterial suspension, agar diffusion, and intensity of biofilm formation, but Se/CAR and Se/AG NCs inhibited the growth of *Pseudomonas oryzihabitans*. The cell growth was decrease by 15–30% during the entire observation period, but the stimulation of biofilm formation by this bacterium was observed for Se/CAR NC. Se/AG NC also had bacteriostatic and antibiofilm effects on the rhizospheric bacterium *Acinetobacter guillouiae*. There was a 2.5-fold decrease in bacterial growth and a 30% decrease in biofilm formation, but Se/CAR NC stimulated the growth of *A. guillouiae*. According to the results of the preliminary field test, an increase in potato productivity by an average of 30% was revealed after the pre-planting treatment of tubers by spraying them with Se/AG and Se/CAR NCs with the same concentration of Se of 0.000625% (6.25 µg/mL) in a final suspension. The obtained and previously published results on the positive effect of natural matrix-based Se NCs on plants open up prospects for further investigation of their effects on rhizosphere bacteria and resistance of cultivated plants to stress factors.

## 1. Introduction

One of the approaches helping to overcome the food shortage in the world is the use of the unique properties of nanotechnology in the agricultural sector [1,2]. The significant potential of some nanomaterials (nanoporous zeolites, nanocapsules, nanosensors, carbon nanotubes, etc.) for protecting the host plant from biotic and abiotic stresses has been recognized [3]. At the same time, such important properties of these materials such as the ability of self-assembly, stability, specificity, the possibility of their microencapsulation, biocompatibility, and safety are still to be exploited [1,2,3,4]. An increase of agricultural crop productivity is achieved, among other things, through biotechnological methods of ensuring plant resistance to various phytopathogens [5,6]. Currently, nanoparticles in plant biotechnology can be used to regulate the synthesis of biologically active substances in the cell cultures of producing plants [7,8]. Metal nanoparticles in sensor devices have found application in agricultural technologies in connection with the detection of phytopathogens [9]. The quantity and quality of products are also increased by optimizing nutrition, and plant protection is enhanced by using various types of nanomaterial-based metal oxides, ceramics, silicates, magnetic particles, quantum dots, polymers, dendrimers, and emulsions [10,11,12], but their environmental safety should be always addressed.

Most of the agents used in agriculture to control phytopathogens have a fungicidal effect but no or weakly negative effect on the phytopathogenic bacteria. Therefore, it is very important to develop new substances, such as NCs, that will control not only phytopathogenic fungi but also bacteria, and, at the same time, will be safe for the environment, in particular for the rhizosphere microorganisms that are very important for plants.

The rhizosphere is a region of the soil close to the plant roots [13] where the most favorable conditions for maintaining bacteria in large quantities are created due to the presence of root exudates used by microorganisms for nutrition. Through their root system, plants release both complex biologically active organic compounds (chemoattractants, vitamins, enzymes, hormones) and simple substances (oligosaccharides, amino acids, alcohols, aldehydes) into the environment [14,15]. Due to the consumption of these compounds and dead root cells, the rhizosphere’s microbial biomass increases rapidly and exhibits high biological activity [16].

The rhizosphere’s microorganisms are an important connecting link in the soil–plant system. They ensure plant growth and development, as well as plant adaptation to stress factors. These microorganisms promote the assimilation and processing of nutrients by plants (for example, they mineralize compounds otherwise inaccessible to plants); regulate their hormonal balance by synthesizing auxins, cytokinins, and gibberellins; and provide direct or indirect protection of plants from phytopathogens through the synthesis of antibiotics, toxins, and hydrolytic enzymes [15,17,18,19]. In addition, they contribute to the protection of plants from abiotic stresses such as dehydration and exposure to heavy metals [20,21], while improving the soil structure.

The rhizosphere species spectrum may depend on the plant species and can change during different phases of a growing season [16,22]. The rhizosphere contains various microorganisms: Gram-positive and Gram-negative bacteria, as well as fungi. These include representatives of the genera *Bacillus*, *Pseudomonas*, *Enterobacter*, *Azotobacter*, *Proteobacteria*, *Bacteroides*, *Azospirillum*, *Agrobacterium*, *Aspergillus*, *Penicillium*, *Klebsiella, Micromonospora*, *Nocardia*, *Streptomyces*, *Xanthomonas*, *Enterobacter*, *Chryseobacterium*, *Flavobacterium*, *Talaromyces*, *Gliocladium*, and *Humicola* [9,15,16].

Rhizospheric microorganisms perform systemic functions in such processes as soil formation and soil organic matter decomposition [23]. Rhizosphere microorganisms are able to utilize a wide range of chemical compounds. Thereby, they participate in the bioremediation of disturbed areas (rhizoremediation) [24]. In addition, the microbial community is one of the most sensitive ecological indicators, which mark various stages of soil restoration, since it is able to quickly adapt to environmental changes and master all the available ecological niches in the ecosystem. Today, industrial environment pollution is a very powerful factor causing the destabilization of natural ecosystems. As a result of such processes, the degradation of fertile soils occurs.

As a result of anthropogenic pollution, the use of pesticides causes significant harm to the microbial community. The use of such substances is increasing every year around the world. Most of the currently used pesticides are chemically synthesized. Although pesticides are sprayed directly onto plants and soil, only about 1% of the sprayed pesticide reaches the target [25]. Pesticides can accumulate in seeds and fruits of cultivated plants [26] and contaminate water bodies [27,28] and soil [29], causing negative impact on its inhabitants [8,30]. In relation to soil microorganisms, pesticides act in three directions: they affect the main processes carried out by microorganisms in the soil, they change the number and species composition of representatives of different taxonomic groups of microorganisms, and they change the species composition and organization of microbial communities [31]. In this regard, it is extremely important to search for environmentally friendly substances used for the recovery of cultivated plants.

Due to the special properties of nanosubstances and their compounds, the delivery of nanoparticles (NPs) to the target is possible using nanocomposites (NCs). Previously, we studied several selenium (Se) NCs composed of natural polysaccharide matrices (AG and CAR) as substances for the recovery of cultivated plants from phytopathogenic bacteria [32,33,34]. It was found in these earlier studies that Se NCs at a concentration of 0.000625% (6.25 µg/mL) in the final suspension had an antibacterial effect against the phytopathogenic bacterium that causes diseases of potato ring rot, *Clavibacter sepedonicus*, and did not negatively affect the viability of potato plants at this concentration. However, their fungicidal effect has not been studied. It is also very important to study their impact on the environment, in particular, on the viability of the soil microbiome. The study presented here continues these studies. The main objectives of the study were to investigate the effects of aqueous suspensions of Se NCs with natural polysaccharide matrices in the already established healing for potatoes (not harming the plant and having an antibacterial effect on the phytopathogen) 0.000625% Se (6.25 µg/mL) concentration on the viability of phytopathogenic fungus *Phytophthora cactorum* and rhizospheric microorganisms isolated from the rhizosphere of plants growing in the Irkutsk region, Russia.

## 2. Materials and Methods

### 2.1. Nanocomposites (NCs)

The following NCs were used in the research: arabinogalactan-based Se NC (Se/AG NC, 5.92% Se), starch-based Se NC (Se/ST NC, 1.46% Se), and kappa-carrageenan-based Se NC (Se/CAR NC, 3.67% Se).

The AG was obtained from Siberian larch (Wood Chemistry Ltd., Irkutsk, Russia). It was additionally purified from impurities and flavonoids [35] by passing it through a polyamide column.

CAR (potassium-sodium salt of sulfated anhydro-polysaccharide) of WR-78 type (CP Kelco ApS, Lille Skensved, Denmark) was used for synthesis of the Se/CAR NC.

ST from potato as a water-soluble reagent (Sigma-Aldrich, Saint Louis, MO, USA) was used for synthesis of the Se/ST NC.

Selenium dioxide (99.8%, Sigma-Aldrich, Saint Louis, MO, USA) as a selenium precursor and L-ascorbic acid as a reductant (99.0%, Sigma-Aldrich, Saint Louis, MO, USA) were used for synthesis of the Se/AG NC and Se/CAR NC.

Available sodium bis(2-phenylethyl)diselenophosphinate [36] as a selenium precursor and hydrogen peroxide water solution (30%, Sigma-Aldrich, Saint Louis, MO, USA) as an oxidant were used for synthesis of the Se/ST NC.

#### 2.1.1. Se/AG NC

Powders of 1.0 g of SeO_2_ and 0.4 g of ascorbic acid were successively added to a suspension of 4.0 g AG in 30 mL of H_2_O with stirring on a magnetic stirrer. The reaction mixture was stirred for 30 min at room temperature. The appearance and gradual deepening of the orange color of the reaction mixture were observed. Then, the reaction mixture was poured into 150 mL of ethanol, and the formed orange precipitate of Se/AG NC was filtered, washed on the filter with ethanol, and dried in air to constant weight.

#### 2.1.2. Se/CAR NC

Due to the slow dissolution of CAR through the stage of its preliminary swelling, 5 g of this polysaccharide was kept in 350 mL of water at room temperature and stirring on a magnetic stirrer for 12 h until complete dissolution. Then, with stirring, aqueous suspension of 0.375 g of SeO_2_ in 5 mL of H_2_O and solution of 0.227 g of ascorbic acid in 5 mL of H_2_O were successively added. A very slow appearance and deepening of the orange color of the reaction mixture were observed. After 24 h, the reaction mixture was poured into 1500 mL of ethanol, the resulting orange Se/CAR NC precipitate was filtered, washed on a filter with ethanol, and dried in air to constant weight.

#### 2.1.3. Se/ST NC

Due to the slow dissolution of ST through the stage of its preliminary swelling, 2 g of this polysaccharide was mixed with 250 mL of water at room temperature under stirring on a magnetic stirrer. Then, the temperature was raised for 10 min until the resulting mixture boiled, cooled to 40 °C, 0.3 g of sodium bis(2-phenylethyl)diselenophosphinate was added with stirring, and the mixture was held at 40 °C for 3 h. Then, 10 mL of concentrated (30%) H_2_O_2_ was added, and the reaction mixture was additionally held at the same temperature for 1 h. Isolation of the Se/ST NC and its purification from the sodium bis(2-phenylethyl)phosphinate byproduct were carried out by pouring the reaction mixture into a fourfold excess of ethanol followed by washing on a filter with the same solvent and air drying to constant weight.

All resulting NCs were well dispersible in water. Their aqueous colloid suspensions were brought to 0.000625% of Se (6.25 µg/mL) and used in the experiments with bacteria and potato. This concentration was selected based on our previous studies of antibacterial activity of similar Se NCs and was effective against the phytopathogenic bacteria *Clavibacter michiganensis* subsp. *sepedonicus* and *C. sepedonicus* in our experiments [37,38]. Taking into account that synthesized in our study arabinogalactan-based Se/AG NC contained 5.92% Se, starch-based Se/ST NC—1.46% Se, and kappa-carrageenan-based Se/CAR NC—3.67% Se, the following concentrations of Se NCs were used to gain 0.000625% of Se (6.25 µg/mL): 105.57 µg/mL for Se/AG NC, 428.08 µg/mL for Se/ST NC, and 170.30 µg/mL for Se/CAR NC.

### 2.2. Fourier-Transform Infrared Spectroscopy (FTIR) of Se NCs

The qualitative functional group analysis of the NCs was performed by FTIR. The FTIR spectra were recorded on the Bruker Vertex 80 spectrometer (Bruker Corporation, Bremen, Germany) in KBr pellets. Measurements were performed in a spectral range from 400 to 3800 cm^−1^.

### 2.3. Optical Absorption in the Ultraviolet and Visible Ranges (UV-Vis) of Se NCs

UV-Vis was used to detect the optical properties of nanocomposites that reflect electronic energy transitions corresponding to the energies of absorbed light quanta in the ultraviolet and visible ranges. Spectra of 0.01% aqueous suspensions of NCs in 10 mm quartz cells were recorded on a UV-1900 UV-Vis Shimadzu Spectrophotometer (Shimadzu, Kioto, Japan).

### 2.4. X-ray Phase Analysis (XPA) of Se NCs

XPA was used to determine the crystalline modification, degree of crystallinity, and crystallite size (mean coherent scattering region) of the Se nanoparticles. XPA of Se NCs was performed on tablets made of compressed Se NC powders using a powder diffractometer Shimadzu XRD-7000 diffractometer (Shimadzu, Kioto, Japan) (Cu-Kα radiation, Ni–filter, 3–35° 2θ range, 0.03° 2θ step, 5 s per point).

### 2.5. Scanning Electron Microscopy Combined with Energy-Dispersive X-ray Microanalysis (SEM/EDXMA) of Se NCs

Images of the surface of the Se NCs powders were obtained using Hitachi TM 3000 scanning electron microscope (Hitachi High-Tech America, Inc., Schaumburg, IL, USA) equipped with an Xflash 4304 SD detector. Percentage of Se in NCs was determined based on the EDXMA data obtained using this scanning electron microscope. Se NCs were adhered to a microscope stage using electroconductive glue and placed into a scanning electron microscope chamber, where they were subjected to electron impact. Atoms of the samples were excited by electron beam, and, thus, emitted X-rays of wavelengths characteristic of each chemical element. Analyzing the energy spectrum of X-ray emissions, we assessed the sample qualitative and quantitative composition.

### 2.6. Transmission Electron Microscopy (TEM) of Se NCs

NCs were dissolved in water. Then, they were applied to grids with formvar substrates, where they were dried. The prepared samples were examined using a LEO 906E transmission electron microscope (TEM) (Carl Zeiss, Oberkochen, Baden-Württemberg, Germany) at an accelerating voltage of 80 kV. Micrographs were taken with a MegaView II camera (Arecont Vision Costar, LLC, Glendale, CA, USA) and processed with Mega Vision software version 4.0 (MegaVision, Santa Barbara, CA, USA).

### 2.7. Phytopathogen

We used the filamentous fungus *Phytophthora cactorum* strain VKM F-985 obtained from the All-Russian collection of microorganisms of the Skryabin Institute of Biochemistry and Physiology of Microorganisms (Russian Academy of Sciences, Moscow, Russia).

The mycelial culture of *P. cactorum* was grown at 27 °C on a glucose-peptone-yeast (GPY) nutrient medium composed of (g/L): glucose—20; peptone—2.0; yeast extract—3.0; K_2_HPO_4_—1.0; KH_2_PO_4_—1.0; MgSO_4_•7H_2_O—0.25; pH 6.0. To prepare solid media, 1.8–2% (m/v) agar was added to nutrient solutions.

### 2.8. Bacterial Strains

Microorganisms isolated from the rhizosphere of plants growing in the oil-contaminated territory of Zalarinsky District (Tyret settlement) of the Irkutsk Region, Russia, were used as soil microbiome microorganisms. After isolation of bacteria, their morphological, cultural, physiological, and biochemical properties were studied. The 16S rRNA gene sequencing was used to determine the species composition [39].

The *Acinetobacter guillouiae* strain was isolated from the rhizosphere of quack grass *Elytrigia repens*. Their short rod-shaped cells are Gram-negative. They formed beige, round, shiny, slightly convex colonies up to 1.2 mm in size with homogeneous structure and soft consistency with a smooth edge. They were aerobes and oxidase and catalase positive.

The *Rhodococcus erythropolis* strain was also isolated from the rhizosphere of *E. repens* quack grass. At an early stage of development, the culture was a rudimentary mycelium, which later broke down into fragments. The fragments then turned into rods and then into cocci. The bacteria are Gram-positive. Their colonies were cream-colored, matte, pasty, and convex, with a rhizoid edge and of rough structure, medium-sized, about 1.5–2.5 mm in size. They were aerobes, oxidase-negative, and had the catalase activity.

The *Pseudomonas oryzihabitans* strain was isolated from the rhizosphere of *Carex hancockiana sedge*. The straight rod-shaped cells were single or connected in twos. They are Gram-negative. When cultivated on solid media, they formed yellow, smooth, shiny, round-shaped, convex, slimy colonies with smooth edges, homogeneous structure and soft consistency. They were aerobes and capable of pigmentation. They were catalase and oxidase positive [40].

To determine the species in pure cultures, the nucleotide sequence of the 16S rRNA gene was analyzed. It showed the following nucleotide sequences: 1136 bp with 98% of similarity to *R. erythropolis*; 1429 bp with 99% of similarity to *P. oryzihabitans*; and 1098 bp with 98% of similarity to *A. guillouiae*. The results revealed the generic affiliation to *Rhodococcus*, *Pseudomonas*, *Acinetobacter*.

The bacteria were cultured in the dark for a day on a solid medium consisting of agar and enzymatic hydrolyzate of beef and on a liquid nutrient medium of a similar composition.

### 2.9. Fungicidal Activity Analysis

We compared radial growth of the fungus *Phytophthora cactorum* strain VKM F-985 on a solid medium in the absence and in the presence of a Se NC at different concentrations. We also used a known fungicide fludioxonil alone capable of inhibiting the growth of the mycelium of a pathogenic fungus for comparison. The method consisted of the following: sterile, melted, and cooled to about 60 °C agar growth medium GPY was mixed with suspensions of the studied Se NC and poured into a sterile Petri dish (20.0 mL). The final concentrations of Se/AG, Se/ST, Se/CAR NCs ranged from 35 to 300 μg/mL in the Petri dishes (Table 1).

After solidification of the medium, they were inoculated by the fungus using 10-day-old cultures of *P. cactorum* grown on agar GPY. The inoculation was done by introducing 5 mm in diameter GPY-agar discs with fungus culture excised from the growing mycelium using a sterile metal punch and then transferred with a sterile scalpel to the center of the Petri dish and incubated in a thermostat at 27 °C. The fungicidal effect was scored by the size of the diameter of the fungus colony on a Petri dish compared to the control without Se NC. A commercial fungicide fludioxonil with a final concentration of 10 μg/mL in the growth media was also used for comparison. Each treatment was carried out in at least four replicates in two independent experiments. The observation period ended when the control Petri dish was filled with mycelium (usually after 12 days). The inhibition (I) of the radial growth (R) of the phytopathogen by Se NCs or fludioxonil was calculated according to [41,42] as a percentage by which the normal radial growth was decreased compared to the control: I (%) = 100 × (R_control_ − R_treatment_)/R_control_. The effective concentration causing 50% of growth inhibition (EC_50_) was determined from dose-relative inhibition curves.

### 2.10. Antibacterial Activity Analysis

To study the bacteriostatic activity of Se NCs against bacteria, a liquid culture of microorganisms was grown in the dark at 26 °C on a shaker (80 rpm) in flasks with a nutrient medium. For all experiments with bacteria, the following NC concentrations were used: 105.57 µg/mL for Se/AG NC, 428.08 µg/mL for Se/ST NC, and 170.30 µg/mL for Se/CAR NC, which corresponds to the Se concentration of 6.25 µg/mL in the final solution. After adding 300 µL of Se NCs per well, the optical density of the suspension was measured immediately and after 2, 4, 24, 28, 48, 48, 52, and 72 h of co-incubation at 595 nm using a BIO-RAD plate spectrophotometer model 680 (Bio-Rad Laboratories, Inc., Hercules, CA, USA). The detection of the Se NC bactericidal effect was carried out using the circle method (agar diffusion) [43]. The influence of Se NCs on the bacteria biofilm formation with concentration of Se of 6.25 µg/mL was studied using the plate method [44] with nine independent observations per each Se NC, control and bacteria.

The obtained data were statistically compared using the nonparametric Mann–Whitney U test.

### 2.11. Field Experiment

Two weeks before planting, tubers of potato variety “Sante”, zoned in the region where the study was carried out, were sprayed with an aqueous Se NC suspension with the following concentrations: 105.57 µg/mL for Se/AG NC, 428.08 µg/mL for Se/ST NC, and 170.30 µg/mL for Se/CAR NC, which corresponds to the Se concentration of 6.25 µg/mL in each NCs in the final solution. Control tubers were sprayed with water. Seed material of all variants was planted in the soil on the field of the experimental plot of the Siberian Institute of Plant Physiology and Biochemistry, Siberian Branch of the Russian Academy of Sciences, (Irkutsk, Russia). Each experimental group contained 30 plants planted in three different plots. The experiment lasted 90 days. The cultivation of plants was carried out with periodic weeding and hilling without additional watering and fertilization, in the extreme continental climate of Eastern Siberia (Irkutsk, Russia). At the end of the growing season, the productivity was assessed for pooled tubers from all 30 plants per plot, and the analysis of the structure of the potato yield was carried out using the nonparametric Mann–Whitney U test. We did not measure the selenium content in tubers treated with NCs, because these tubers were not intended to be used for human or animal consumption, but as planting material. Therefore, we were mainly interested in a long-term effect of exposure to seed material, which could manifest itself during the growing season and affect productivity.

## 3. Results

### 3.1. Physicochemical Characterization of Se NCs

#### 3.1.1. FTIR Spectroscopy of Se NCs

**Figure 1:**
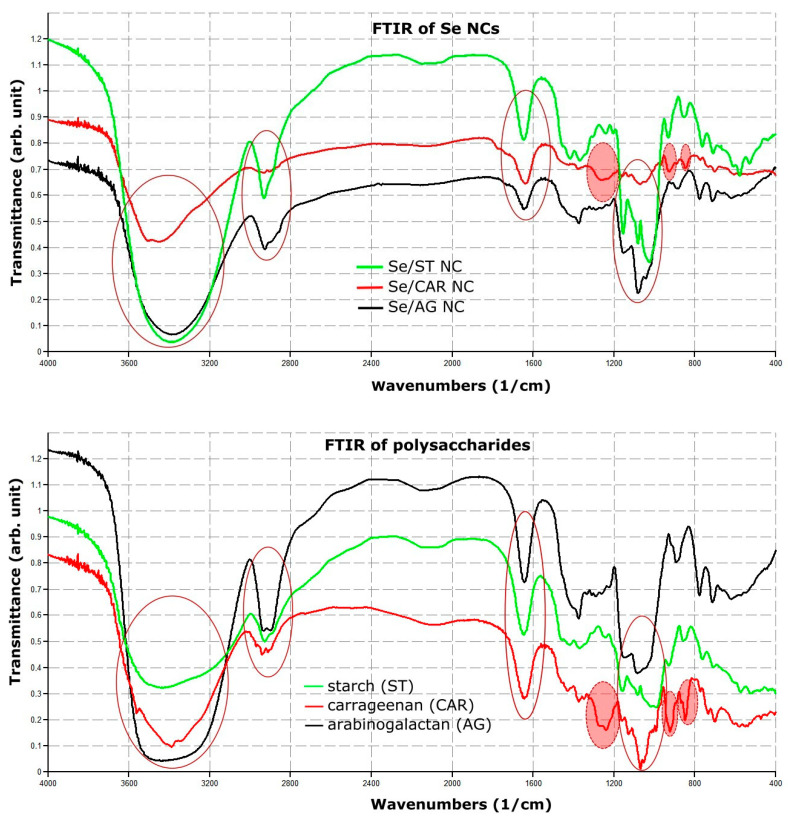
Fourier-transform infrared (FTIR) absorption spectra of all Se NCs and their polysaccharides. Continuous ovals depicted four intense IR absorption regions that were almost the same for all Se NCs in our study. Dotted solid red ovals depicted three additional IR absorption regions specific for CAR and Se/CAR NC.

FTIR absorption spectra of all Se NCs and their polysaccharides were largely similar (Figure 1 and Figure S1).

There were four intense IR absorption regions that almost coincided with each other: the dominant broad absorption in the region of 3000–3800 cm^−1^, as well as 2850–3000 cm^−1^, 1636 cm^−1^, and 1000–1200 cm^−1^ (Figure 1, depicted by continuous ovals). There were also three additional pronounced IR absorption regions for CAR and Se/CAR NC: a compound composite band at 1230–1260 cm^−1^, as well as two bands at 928 cm^−1^ and 845 cm^−1^ (Figure 1, depicted by dotted solid red ovals).

#### 3.1.2. UV-Vis Spectroscopy of NCs

**Figure 2:**
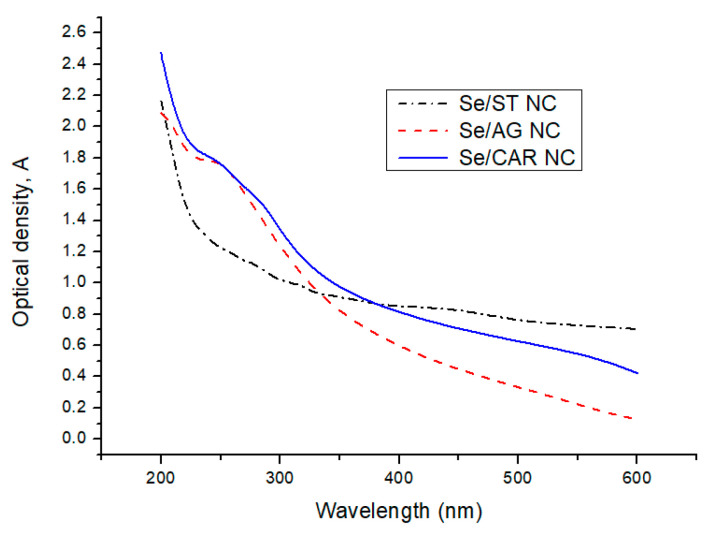
UV-Vis optical density spectra of SeNPs.

All NCs demonstrated broad decaying optical absorption in the 200–600 nm wavelength range of light (Figure 2). Moreover, there was a prominent absorption region in the range of 225–230 nm for Se/AG NC. In the case of Se/CAR NC, there were three discernible absorption regions in the ranges of 225–260, 260–325, and 500–600 nm. There were also two weakly pronounced regions in the ranges of 250–350 and 400–500 nm for Se/ST NC.

#### 3.1.3. XPA of Se NCs

**Figure 3:**
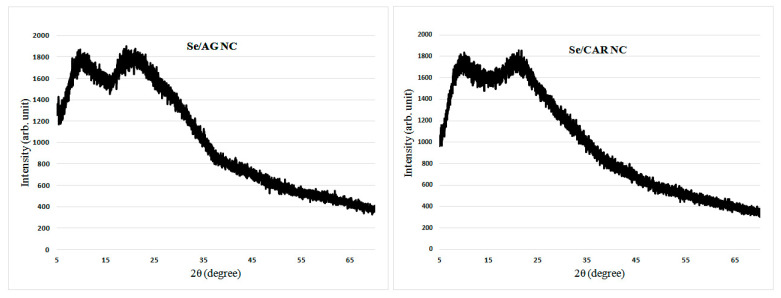
Typical diffractograms of Se/AG and Se CAR NCs.

Diffractograms represent XPA spectra and are graphs of the dependence of the scattered radiation intensity on the scattering angle. The diffractograms of Se/AG and Se/CAR NCs were typical with no pronounced reflexes and demonstrated only wide halos from polysaccharide matrices (Figure 3).

**Figure 4:**
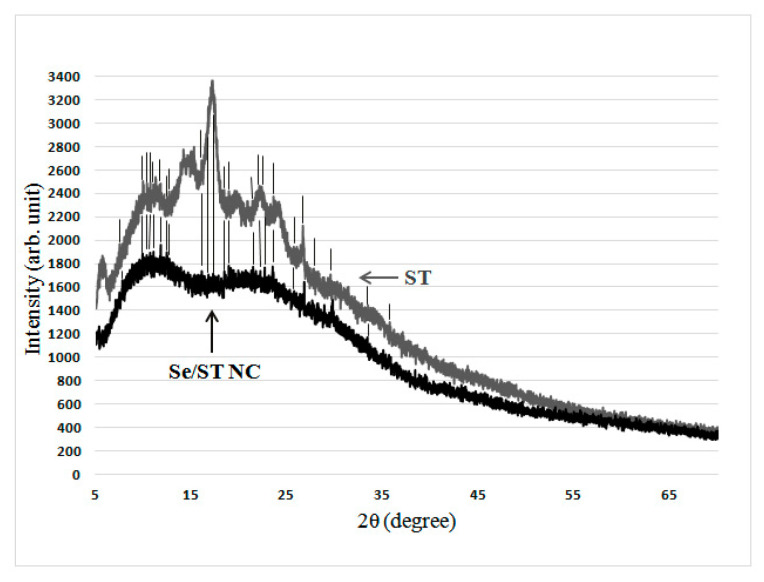
Diffractogram of the Se/ST NC and the original ST (vertical lines depict the Se/ST NC reflexes, which coincide with those for the original ST).

However, in the case of Se/ST NC, its diffractogram still showed weak reflexes, which coincided with a number of reflexes of the original ST used for the synthesis of Se/ST NC (Figure 4, coincidence is marked by vertical lines).

#### 3.1.4. SEM/EDXMA of Se NCs

**Figure 5:**
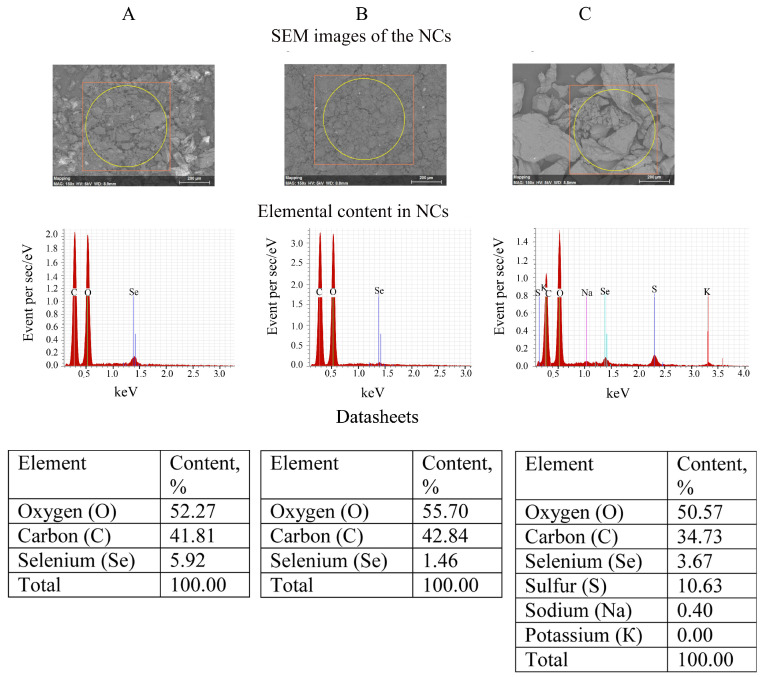
Results of energy-dispersive X-ray spectroscopy microanalysis (EDXMA) of Se/AG (**A**), Se/ST (**B**), and Se/CAR (**C**) NCs.

SEM images of NCs powders showed them agglomerated into irregular micrograin structures. While the EDX Se/AG and Se/ST NCs spectra obtained with the SEM microscope contained only X-ray emission lines of carbon, oxygen, and selenium, the EDX spectrum of Se/CAR NC, in addition to the emission of these three elements, also included X-ray emission lines of potassium, sodium, and sulfur. Finally, 3.2 g of Se/AG NC and 4.7 g of Se/CAR NC were obtained as an orange powder containing 5.92% and 3.67% of Se, respectively, according to the EDXMA (Figure 5). Regarding Se/ST NC, 1.9 g were received in total in the form of a pale gray powder containing 1.46% of Se according to the EDXMA (calculated approximately by considering a negligible contribution of the lightest hydrogen to the total percentage of elements, which cannot be detected by the EDXMA method).

#### 3.1.5. TEM of NCs

**Figure 6:**
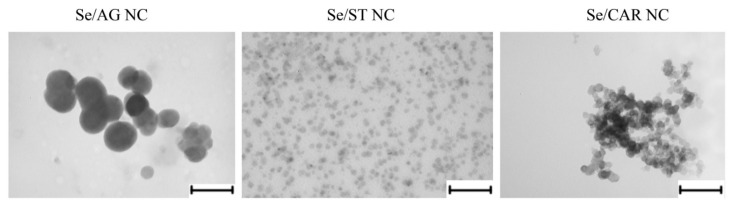
Photos of the Se NCs obtained using a transmission electron microscope LEO 906E. Scale bar = 200 nm.

TEM images showed rounded Se nanoparticles, which, depending on the polysaccharide matrix of NCs, differed significantly in size and were associated with each other in different ways (Figure 6). The smallest sizes (~20–40 nm) had Se nanoparticles, which were evenly, practically without associations with each other, distributed in ST matrixes. The largest (~100–180 nm) were the Se nanoparticles in AG matrices. In this case, Se nanoparticles were already associated in sligthly elongated clusters. Se nanoparticles, which were strongly associated in highly elongated chain clusters in CAR matrixes, had an intermediate size (~40–70 nm).

### 3.2. Fungicidal Effect of NCs

The assessment of the resistance of *P. cactorum* to suspensions with putative fungicidal properties was carried out on agar nutrient medium. We studied the susceptibility of the fungus to the Se NCs and, for comparison, to the commercial fungicidal fludioxonil. Fludioxonil is one of the most effective fungicides against diseases caused by a number of phytopathogenic fungi, including *Phytophthora* spp. [45]. In laboratory conditions, the assessment of the resistance of phytopathogenic fungi to this fungicide is carried out at its concentrations from 0.1 to 10 μg/mL [46].

Screening for the presence of antifungal properties of the Se NCs introduced into the agar medium for the cultivation of the fungus was carried out in their concentration range from 35 to 300 μg/mL (Table 1). The inhibition (I) of fungal growth was calculated as a percentage, taking into account that the quantitative characteristic of the absence of inhibition is expressed as 0% (Table 1).

The EC_50_ value was calculated as the drug concentration at which the radial growth of the fungus colony was decreased by 50% relative to the non-fungicidal control. Despite the fact that EC_50_ is a rather formal indicator based on the assumption of an inverse linear dependence of the colony diameter of the mycelial test object on the concentration of the fungicide, this indicator is able to characterize the relative fungicidal activity of different drugs when their concentration in the nutrient medium of the fungus is close to a concentration inhibiting radial growth of colonies by two times.

Two thirds of the studied Se NCs at different concentrations were characterized by an inhibition value of more than 20%, and half (18 out of 36 combinations in Table 1) inhibited the growth of pathogen colonies by at least 30% (Table 1). The minimum EC_50_ value of 58 μg/mL was observed for the Se/ST NC.

There was an increase in the antifungal ability with the increase in concentration and from day 4 to day 10 (Table 1), except Se/AG NC of 200 μg/mL, Se/ST NC of 35 and 70 μg/mL, and Se/CAR NC of 39 μg/mL. However, in all these cases, the decrease in the inhibition value was no more than 7% by the 10th day of cultivation. Thus, the studied fungicidal preparations significantly inhibited the growth of *P. cactorum* mycelium (up to 60% for the Se/AG NC) during the growth of the fungus, and there was practically no loss of their fungicidal properties.

### 3.3. Antibacterial Effect of Se NCs

#### 3.3.1. Bactericidal Effect

**Figure 7:**
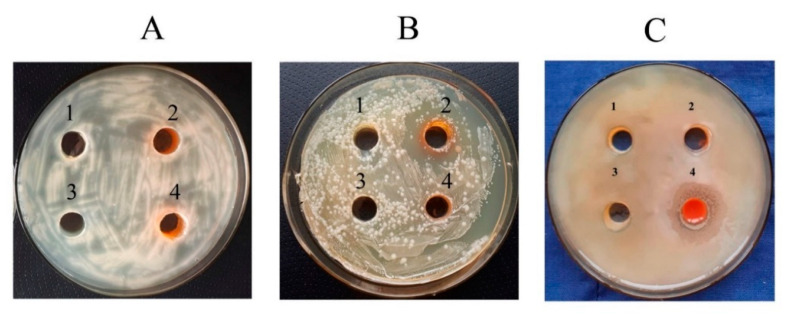
Effect of the Se NCs on the growth of rhizosphere bacteria (with 105.57 µg/mL for Se/AG NC, 428.08 µg/mL for Se/ST NC, and 170.30 µg/mL for Se/CAR NC, which corresponds to the Se concentration of 6.25 µg/mL in the final solution): (**A**) *R. erythropolis,* (**B**) *A. guillouiae*, (**C**) *P. oryzihabitans*. **1**—control, **2**—Se/AG NC, **3**—Se/ST NC, **4**—Se/CAR NC.

The studied Se NCs did not affect *A. guillouiae* and *R. erythropolis* bacteria (Figure 7A,B). Inhibition (a pronounced zone of no growth) was found only for *P. oryzihabitans* and only under the influence of Se/CAR NC with the concentration of 170.30 µg/mL (Figure 7C: 4). Its size reached 12 ± 0.3 mm (Figure 7C: 4).

#### 3.3.2. Bacteriostatic Effect

**Figure 8:**
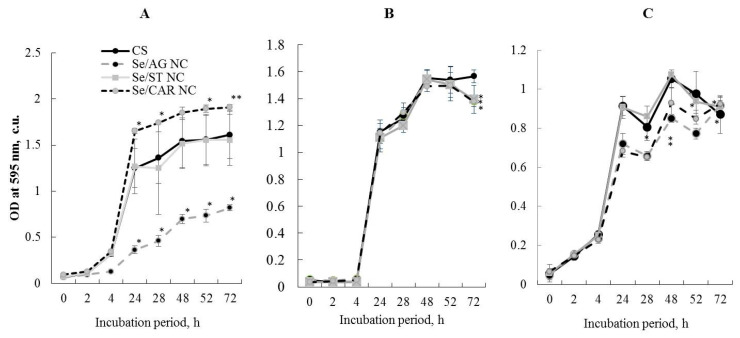
Effect of the Se NC treatments (with 105.57 µg/mL for Se/AG NC, 428.08 µg/mL for Se/ST NC, and 170.30 µg/mL for Se/CAR NC, which corresponds to the Se concentration of 6.25 µg/mL in the final solution) on the growth dynamics of rhizosphere bacteria *A. guillouiae* (**A**), *R. erythropolis* (**B**), and *P. oryzihabitans* (**C**). CS—control samples; * *p* < 0.01, ** *p* < 0.05 (in comparison with CS at each respective time point) based on 9 independent observations per each Se NC, control and bacteria.

When studying the presence of the Se NC bacteriostatic effect on rhizosphere bacteria, typical curves of bacterial growth were comparable with those observed in the control samples (Figure 8). The experiments demonstrated the absence of the bacteriostatic effect of Se/ST NC (with concentration of 428.08 µg/mL) on the studied microorganisms (Figure 8). For each Se NC, control, and each bacteria, nine independent replicates were used. The means and standard deviations were calculated and compared. No bacteriostatic effect of Se/CAR NC (with concentration of 170.30 µg/mL) on the bacteria *A. guillouiae* and *R. erythropolis* was also revealed (Figure 8A,B). Moreover, it stimulated the growth of *A. guillouiae* (Figure 8A). Se/CAR NC inhibited the growth of the bacteria *P. oryzihabitans* (Figure 8C). The decrease began on the first day of the experiment and ranged from 15 to 30% during the entire observation period.

#### 3.3.3. Anti-Biofilm Formation Effect

**Figure 9:**
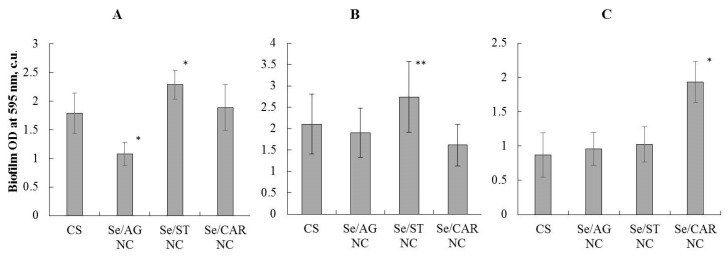
Effect of the Se NC treatments (with 105.57 µg/mL for Se/AG NC, 428.08 µg/mL for Se/ST NC, and 170.30 µg/mL for Se/CAR NC, which corresponds to the Se concentration of 6.25 µg/mL in the final solution) on the biofilm formation of rhizosphere bacteria *A. guillouiae* (**A**), *R. erythropolis* (**B**), and *P. oryzihabitans* (**C**); * *p* < 0.01, ** *p* < 0.05 (in comparison with CS) based on 9 independent observations per each Se NC, control and bacteria.

The concentration of all Se NCs used to study their anti-biofilm formation effect corresponded to the Se concentration of 6.25 µg/mL in the final solution. The Se/AG NC (with a concentration of 105.57 µg/mL) significantly reduced the biofilm formation of the bacterium *A. guillouiae* by 30% compared to the control (Figure 9A). It did not affect the other studied bacteria significantly. No negative effect of Se/ST NC (with concentration of 428.08 µg/mL) on the biofilm formation of rhizosphere bacteria was revealed (Figure 9), it even stimulated the biofilm formation of *A. guillouiae* and *R. erythropolis* (Figure 9A,B). No effect of Se/CAR NC (with concentration of 170.30 µg/mL) on the biofilm formation under study was found in *A. guillouiae* and *R. erythropolis*. This NC even stimulated the formation of biofilms in *P. oryzihabitans* (Figure 9C).

### 3.4. Effect of Nanocomposites on Potato Productivity in the Field Experiment

**Figure 10:**
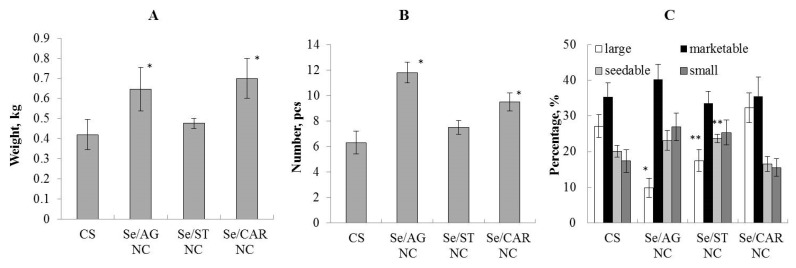
Effect of the Se NC treatments (with 105.57 µg/mL for Se/AG NC, 428.08 µg/mL for Se/ST NC, and 170.30 µg/mL for Se/CAR NC, which corresponds to the Se concentration of 6.25 µg/mL in the final solution) on the average weight of potatoes (**A**) and number of tubers (**B**) per plant, and crop structure (**C**). Large tubers weighed 150 g or more; commercial (marketable)—85–150 g; seed—50–80 g; small—less than 50 g; * *p* < 0.01, ** *p* < 0.05 (in comparison with CS) based on 3 plots per each Se NC and control.

Based on the results of the field experiment, we analyzed such indicators as the average mass of a potato tuber and the number of tubers per plant, and carried out a tuber analysis (Figure 10). Se/AG NC (with concentration of 105.57 µg/mL) had the maximum stimulating effect. In comparison with the control (CS), it significantly stimulated an increase in the average tuber mass (Figure 9A) and the number of tubers (Figure 10B) per plant. It did not have a positive effect on the structure of the yield and even reduced the number of large tubers. (Figure 10C).

Se/ST NC (with concentration of 428.08 µg/mL) had no significant effect on potato productivity. It did not affect the average mass of tubers (Figure 10A) and their number (Figure 10B) per plant. However, under the influence of Se/ST NC, the number of seed tubers in the yield structure increased in comparison with the control (Figure 10C).

Se/CAR NC (with concentration of 170.30 µg/mL) significantly stimulated an increase in the number of tubers (Figure 10B) and the average tuber mass per plant (Figure 10A) in comparison with the control. There was no significant effect of Se/CAR NC on the yield structure (Figure 10C).

## 4. Discussion

For the synthesis of Se nanoparticles in polysaccharide matrices AG and CAR, the well-proven reduction reaction in an aqueous suspension of Se dioxide with a biogenic biocompatible reducing agent, ascorbic acid, was used [47]. In the synthesis of Se nanoparticles in ST, a new [38,48,49] oxidation reaction in an aqueous suspension of available sodium diselenophosphinate [36] with hydrogen peroxide was used.

The obtained similar FTIR spectra of all synthesized NCs and their polysaccharides, largely coinciding with each other, can be explained by the common polysaccharide nature of biopolymer matrices in these NCs (Figure 1 and Figure S1). Thus, the prevailing broad absorption in the range of 3000–3800 cm^−1^, as well as 1636 cm^−1^, refers to hydroxyl groups in macromolecules of polysaccharides, the IR absorption region of 2850–3000 cm^−1^ refers to the CH bonds, and the group of signals in the region 1000–1200 cm^−1^ refer to ether C–O–C bonds (Figure 1, depicted by a continuous oval) [50]. In addition to these IR absorption bands common to all NC bands, there was also a region from 1230–1260 cm^−1^ for Se/CAR NC, which belongs to the sulfoether groups of carrageenan, a band with a maximum of 928 cm^−1^, which belongs to the C–O–C ester bond of the anhydrogalactose carrageenan cycle, as well as the 845 cm^−1^ band, which belongs to the sulfo groups of galactose-4-sulfate carrageenan units [50]. The absence of pronounced changes in the FTIR absorption bands between polysaccharides and their corresponding Se NCs indicates that polysaccharide matrices are not involved in the redox processes of the synthesis of Se NCs. A slight decrease in the intensities of the absorption bands of the ether and sulfo groups in the range of 800–1260 cm^−1^ in Se/CAR NC can be explained by their broadening due to the predominant interaction of these negatively charged and polarized atomic groups with the surface of the formed Se nanoparticles.

The broad long-wavelength regions observed in the optical absorption spectra of all obtained NCs in the range of 400–600 nm (Figure 2) can be attributed to overlapping individual size-dependent plasmon resonances of polydisperse semiconductor Se nanoparticles, and the higher-energy, short-wavelength optical absorption in the ultraviolet 200–350 nm range (Figure 2) can be attributed to the overlapping individual size-related exciton excitations in polydisperse semiconductor quantum dots of elemental Se [51].

The absence of pronounced reflexes in the X-ray diffraction patterns indicated that Se NCs based on AG and CAR were X-ray amorphous (Figure 3). The presence of weakly expressed reflexes in the case of Se/ST NC, as well as their coincidence with a number of reflexes of the original ST used for the synthesis of Se/ST NC, can be explained by a certain inheritance in the structure of the obtained Se/ST NC of regions of high ordering of the structure of the original ST. However, it can be seen that this ordering of the original ST in Se/ST NC was far from completely preserved, but, on the contrary, was significantly lost in Se NCs after their synthesis. This was indicated by the disappearance of the pronounced maxima of X-ray reflection from the ordered regions of the original ST in the obtained Se/ST NC (Figure 4). As a result, in the synthesized Se/ST NC, one can see an incomplete series of weakly expressed reflexes of the original ST against the background of an X-ray amorphous halo of an already significantly disordered ST matrix (Figure 4). Thus, based on the simultaneous group coincidence of a whole series of reflexes, it can be stated that these Se/ST NC reflections relate specifically to the ST phase in the Se/ST NC structure, while the Se phase is in an X-ray amorphous state.

Despite the X-ray amorphous structure of Se NCs in all the obtained NCs, visually, by the different color of NCs powders, it can be assumed that the orange Se/AG NC and Se/CAR NC contain nanoparticles of the red modification of elemental Se, while the pale gray Se/ST NC contain nanoparticles of the X-ray amorphous black or most likely gray modification of elemental Se.

Previously, we studied the biological activity of Se NCs in natural matrices in order to create effective and environmentally friendly agents on their basis for the recovery of potatoes from bacterial phytopathogens [32,33,34,38], but their potential effects on microbiome were insufficiently studied and addressed in the presented study.

*P. cactorum* is a widely specialized phytopathogen, one of the causative agents of diseases of cultivated plants (e.g., strawberries, alfalfa) widespread in Europe and Asia, including Russia [52,53]. The causative agent of the disease is typical soil-dwelling pathogens that overwinter in the form of mycelium and oospore in the tissues of affected plants. They are also able to persist in plant debris and in the soil in the form of oospores. Infection is carried out by zoospores in spring, when they germinate and penetrate the plant through the epidermis. In infected plants, the fungus forms a delicate mycelium. Conidia form on it, massively infecting plants throughout the growing season. Symptoms of the disease appear on all plant organs: berries, buds, flowers, and inflorescences. In years with high humidity, the tops of the stems and growth points are affected.

The first symptoms are found on the roots of plants in late autumn. On terrestrial organs, they do not appear until spring. In the spring, infected plants are lagging behind in development. Later, the lowest leaves of the rosettes turn yellow, wither, and dry out completely. The damage quickly spreads to neighboring plants. Bushes in the area of the outbreak quickly die. When plucked, damaged plants break off, leaving the main part of the false stem and root in the soil. Most often, plants die during flowering and fruit formation [54]. The intensive development of the pathogen negatively affects the qualitative and quantitative indicators of productivity and reduces the resistance of plants to unfavorable environmental factors. It is especially dangerous in years with high air humidity [55]. Due to the increased risk to the productivity of cultivated plants (for example, infection causes the death of up to 40% of strawberry plants), the development of new effective drugs against *P. cactorum* is urgent. To regulate the numbers of this pathogen, other researchers have used NCs based on silver, sulfur, and tin oxides. According to their results, a number of NCs had an anti-fungal effect [56]. In our studies, it was found that the studied nanocomposites significantly inhibited the growth of *P. cactorum* mycelium (up to 60% for Se/AG NC), during the growth of the fungus there was practically no loss of the fungicidal properties of the preparations (Table 1) that encouraged us to assume the absence of noticeable utilization or biodegradation of the target substance.

In this research, for a comprehensive NC study with the aim of their subsequent use as agents for the recovery of potatoes, we studied their effect on environmental objects, in particular, on representatives of soil microflora. The objects of our research were three non-spore-forming obligate aerobic bacteria belonging to the species *A. guillouiae*, *R. erythropolis*, and *P. oryzihabitans*. We studied different bacteria for a more complete assessment of the Se NC effect on the different representatives of the rhizosphere microbiota. Some species of the studied microorganisms, for example, *R. erythropolis* are capable of performing useful functions in the soil, namely, the biodegradation of hydrocarbons and soil bioremediation [57,58]. In addition, *R. erythropolis* were shown to have the phytostimulating activity. When the seeds were treated with *R. erythropolis* microorganisms, the germination of seeds of oil-bearing radish *Raphanus sativus* L. under conditions of the oil pollution was shown to increase by 25% relative to the control; the root length increased by 50%; the height of the aboveground part and its weight increased by 40%. That indicates a decrease in the negative oil effect on the plant and the presence of phytoprotective properties in *R. erythropolis* [59].

We previously obtained results on the studied Se NC effect on potato plants in vitro and the causative agent of potato ring rot (*Clavibacter sepedonicus*). Se/AG NC with 6.4% of Se was shown to have bacteriostatic and antibiofilm effects [38]. It also was shown to have a positive effect on potato plants, enhancing their immune status by increasing the content of reactive oxygen species (ROS) and increasing the peroxidase activity [32,33,34]. After plant treatment with Se/AG NC, Se was not detected in potato tissues [38]. Se/ST NC with 12.0% Se had weak bacteriostatic and antibiofilm effects to *C. sepedonicus*. However, the treatment with this NC stimulated potato growth and increased the number of leaves both infection-free and infected [34]. Se/CAR NC with 2.0% Se did not suppress the growth of *C. sepedonicus* and their ability to form biofilms. This NC had a positive effect on potato plants in vitro: it stimulated the growth and increased the number of leaves even in plants infected with *C. sepedonicus* [33]. In addition, Se/CAR NC stimulated the length and weight of seedlings during the potato tuber germination. Se remained in insignificant amounts in both infected and infection-free plant tissues after treatment with Se/CAR NC; the Se content was 0.01% and 0.03% of the air-dry mass of the plant, respectively [33]. Despite the available promising data on the NC biological activity, it is known from the literature that pesticides in their targeted use enter the environment in large quantities and cause significant harm to its inhabitants [23,27,28,29,30]. In this regard, we investigated the Se NC effect on the soil bacteria viability.

Data on the antibacterial effect of nanocomposites on rhizosphere bacteria are summarized in Table 2. They have shown that the investigated Se NCs have mostly no pronounced antibacterial effect, but there were only some minor effects for some Se NCs and some bacteria. For example, the bactericidal effect was observed only in *P. oryzihabitans* under the influence of Se/CAR NC (Figure 7). Se/AG NC inhibited the growth of this bacteria (Figure 8, C), as well as another Gram-negative bacteria *A. guillouiae* by more than twofold as compared to the control (Figure 8A) starting from the fourth hour of the co-incubation and continuing throughout the entire observation period. Perhaps this effect is associated with the biological properties of AG, which is a matrix for this NC [37]. A small but significant stimulation of *A. guillouiae* growth was observed under the influence of Se/CAR NC. No antibacterial action of Se NCs was found against the Gram-positive bacterium *R. erythropolis* (Table 2). Probably, its resistance to the Se NCs is associated with the morphology of its cell wall. It is known that Gram-positive bacteria, which include *R. erythropolis*, have a thicker cell wall compared to the thin peptidoglycan cell wall of Gram-negative bacteria [60], which include *P. oryzihabitans* and *A. guillouiae*. In addition, the observed effects can be associated with various enzymes capable of degrading substrates used for food, as well as other substances synthesized by bacteria. *R. erythropolis*, as a microorganism inhabiting the soil, has a high biological activity: it belongs to bioremediants, is able to reduce the negative effect of hydrocarbons on plants [61], and has a phytostimulating effect due to the production of phytohormones, surfactants, and biosurfactants [59]. Possibly, the studied rhizosphere bacteria are highly resistant to the action of various stress factors also due to the presence of plasmids in their cells [62].

The polysaccharide itself can also have biological activity [63]. In addition, of course, this can be related to the size of the Se NCs. A significant characteristic of the successful existence of most bacteria important for agriculture and bioremediation is their ability to form biofilm [64,65]. Se/CAR NC stimulated the formation of biofilms in *P. oryzihabitans* (Figure 9C). Se/AG NC reliably reduced the biofilm formation of the bacterium *A. guillouiae* by 30% compared to the control (Figure 9A).

Preliminary data from the field experiment demonstrated a positive effect of some of the studied Se NCs on the productivity and yield structure of potatoes. The greatest effect of increasing the number of tubers most demanded for nutrition and agriculture (marketable and seed tubers) resulted from the spraying of the pre-planting seeds with Se/CAR NC (Figure 10). The number of such tubers increased by 40% in comparison with the control. Se/AG NC also boosted productivity. Analyzing the structure of the yield, it was found that under the influence of Se/AG NC, the number of marketable and seed tubers increased by 2.5 times, but the number of small tubers was also larger compared to the control (Figure 10). The effect of stimulating the growth, development, and productivity of cultivated plants by nano-selenium is well known. Most of the available published data on nano-selenium indicate its positive effect on plants. For example, it was shown that exogenous spraying by nano-selenium increased the antioxidant potential of basil *Ocimum basilicum* L. [66] and enhanced the growth of *Nicotiana tabacum* L. [67] and *Arachis hypogaea* L. [68]. It was suggested that an increase in the plant growth under influence of nano-selenium occurs due to an increase in photosynthesis [69]. The change in the fatty acid profile of lipids in plant cells under the influence of nano-selenium has also been shown [70]. In addition, it was revealed that nano-selenium affects the activity of antioxidant enzymes in various plant organs—nitrate reductase in leaves and peroxidase in roots [71]. Spraying the plants with a nano-selenium suspension improved growth and increased yields of rice, radish, and corn, and accelerated the growth of lettuce. It was revealed that nano-selenium enhanced the resistance of tomato plants to biotic stress (e.g., Alternaria leaf blight, nematodes, etc.) [72], and enhanced the resistance of strawberry to salt and also increased its productivity [73]. The increased plant resistance to stress is explained by the induction of the activity of such enzymes as superoxide dismutase, ascorbate peroxidase, glutathione peroxidase, phenylalanine ammonia lyase in leaves and glutathione peroxidase in fruits. In addition, the content of chlorophyll *a* and *b* increased in the leaves, and the amount of vitamin C, glutathione, phenols, and flavonoids increased in fruits [73].

## 5. Conclusions

Thus, the effects of the three Se NCs in natural polysaccharide matrices on the viability of phytopathogenic fungus and rhizosphere bacteria have been shown. It was found that Se NCs had a pronounced fungicidal activity comparable to the effectiveness of fludioxonil used in agriculture as an active ingredient in pesticides. The maximum anti-fungal effect against *P. cactorum* was found for Se/AG (up to 60% at 300 μg/mL) and Se/CAR NCs (up to 50% at 234 μg/mL). The nature of the polysaccharide matrix was revealed to affect the Se NC antibacterial effects to rhizosphere bacteria. Se/AG NC was shown to have the bacteriostatic and antibiofilm effects on the Gram-negative bacterium *A. guillouiae*. Se/AG NC reduced the growth of these bacteria by 2.5 times and suppressed biofilm formation by 30% compared to the control. This result can be important for clinical medicine because this bacterium, in addition to being present in the rhizosphere, is a conditionally pathogenic microorganism for humans. *A. guillouiae* can cause human nosocomial infections including bacterial pneumonia and other respiratory infections that are resistant to antibiotic treatment [74]. According to the obtained results, Se/ST NC is a safe substance for the studied rhizosphere bacteria. Se/CAR NC showed the presence of bactericidal and bacteriostatic activity against the bacteria *P. oryzihabitans*. The size of the growth inhibition zone reached 12 mm, the growth of *P. oryzihabitans* started to slow down on the first day of the experiment and decreased by 15–30% during the entire observation period, while the stimulation of biofilm formation by this bacterium was noted. The new data obtained and previously published results on the positive effect of these Se NCs on plants [33,34] open prospects for further research on the soil application of Se/ST NC in combination with rhizosphere bacteria to increase the resistance of cultivated plants to stress factors.

The preliminary data from the field experiment showed an average increase of almost 30% in the productivity of potatoes subjected to the pre-planting treatment by Se/AG and Se/CAR NCs compared to the control. The results presented in this article and described by us earlier [38] characterize Se NCs based on natural polymer matrices as promising and environmentally safe agents for regulating the number of phytopathogenic organisms, and at the same time, having a positive effect on potato productivity.

## Figures and Tables

**Table 1:** Fungicidal activity of selenium (Se) nanocomposites (NCs) against fungus *Phytophthora cactorum* VKM F-985.

Ingredient	Concentration, μg/mL **	Inhibition Value, *I*, % * at Day			EC_50_, µg/mL at Day 4
		4	7	10	
Se/AG NC	50 (3.0)	19	20	26	132 (7.81)
	100 (5.9)	25	33	34	
	200 (11.8)	50	47	43	
	300 (17.8)	56	60	60	
Se/ST NC	35 (0.5)	6	17	11	281 (4.10)
	70 (1.0)	12	23	21	
	139 (2.0)	19	33	40	
	208 (3.0)	37	37	47	
Se/CAR NC	39 (1.4)	0	13	9	205 (7.52)
	78 (2.9)	19	20	23	
	156 (5.7)	25	30	30	
	234 (8.6)	50	47	49	
Fludioxonil	10	46	18	12	10.9

* Only the average values are presented, but the standard deviations did not exceed 0.03 of the presented values. ** Se concentration is presented in brackets.

**Table 2:** Summary data on the antibacterial effect of Se NCs on rhizosphere bacteria in the study.

Effect	NC	*A. guillouiae* (Gram-Negative)	*R. erythropolis* (Gram-Positive)	*P. oryzihabitans* (Gram-Negative)
growth (circle method)	Se/AG	none	none	none
	Se/ST	none	none	none
	Se/CAR	none	none	reduced
growth dynamics (optical density of the suspension)	Se/AG	reduced	none	reduced
	Se/ST	none	none	none
	Se/CAR	stimulation	none	reduced
biofilm formation (plate method)	Se/AG	reduced	none	none
	Se/ST	none	none	none
	Se/CAR	none	none	stimulation

## Data Availability

All data can be found in this paper or in papers cited here.

## Supplementary Materials

The following are available online at https://www.mdpi.com/article/10.3390/nano11092274/s1, Figure S1: Fourier-transform infrared spectroscopy (FTIR) of all Se NCs and their polysaccharides in this study.
