# Supplementary material for: Effect of Natural Polysaccharide Matrix-Based Selenium Nanocomposites on Phytophthora cactorum and Rhizospheric Microorganisms
Source: Nanomaterials (Basel). 2021 Sep 1;11(9):2274. doi: 10.3390/nano11092274 (PMC8466319; doi:10.3390/nano11092274)
Supplement: Supplementary file 1 [file nanomaterials-11-02274-s001.zip › nanomaterials-1314331-supplementary.pdf]

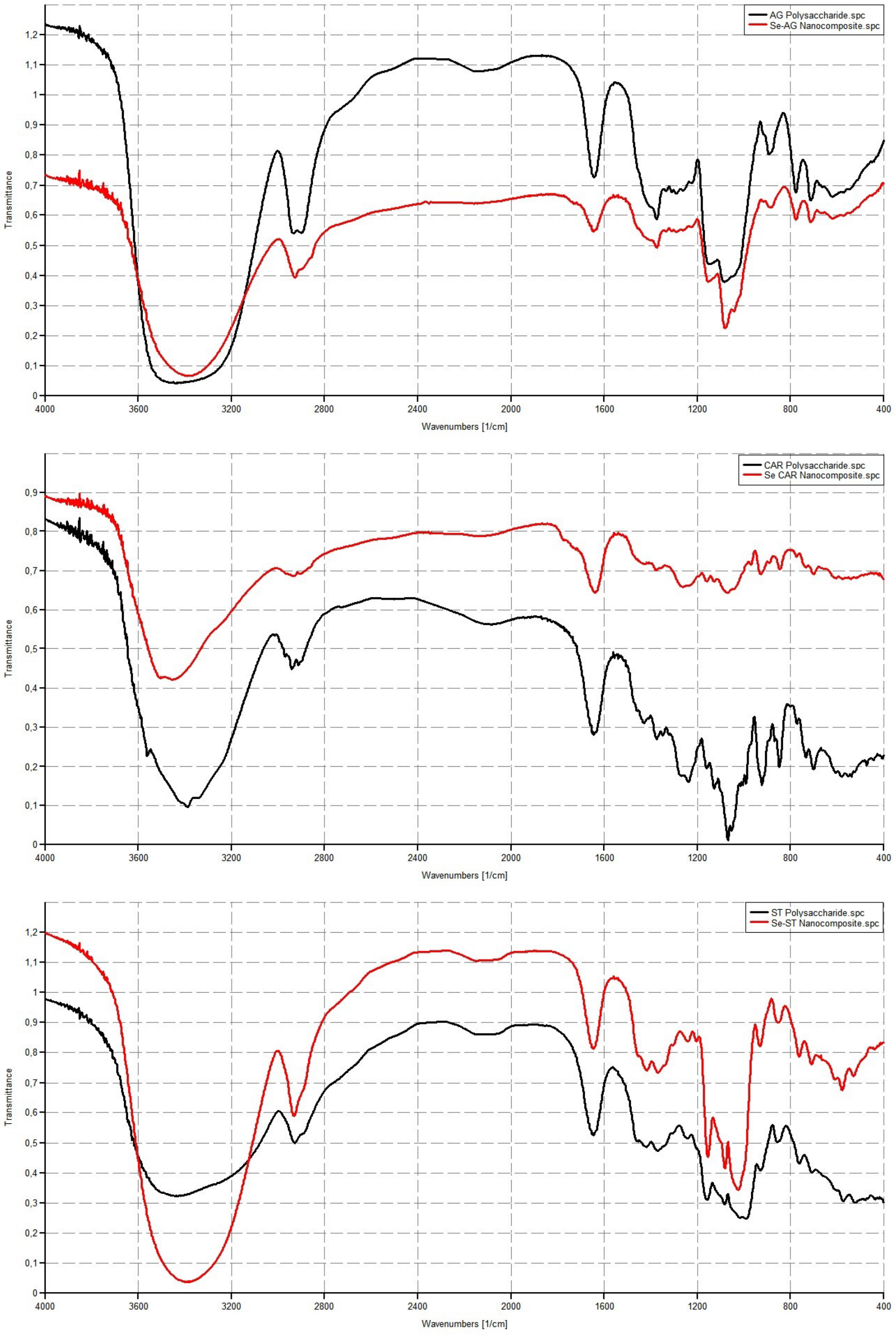

Figure S1: Fourier-transform infrared spectroscopy (FTIR) of all Se NCs and their polysaccharides in this study.
